# The role of Sociodemographic factors on goal achievement in a community-based diabetes prevention program behavioral lifestyle intervention

**DOI:** 10.1186/s12889-021-11844-z

**Published:** 2021-10-02

**Authors:** Susan M. Devaraj, Jenna M. Napoleone, Rachel G. Miller, Bonny Rockette-Wagner, Vincent C. Arena, Chantele Mitchell-Miland, Mohammed Bu Saad, Andrea M. Kriska

**Affiliations:** 1 Department of Epidemiology, University of Pittsburgh Graduate School of Public Health, 5135 Public Health, 130 De Soto Street, Pittsburgh, PA 15261 USA; 2 Department of Biostatistics, University of Pittsburgh Graduate School of Public Health, Pittsburgh, PA USA

**Keywords:** Diabetes mellitus, Metabolic syndrome, Weight loss and weight gain, Exercise, Healthcare disparities, Health disparities, Education, Income

## Abstract

**Background:**

The Diabetes Prevention Program (DPP) behavioral lifestyle intervention was effective among a diverse sample of adults with prediabetes. Demonstrated effectiveness in translated versions of the DPP lifestyle intervention (such as Group Lifestyle Balance, DPP-GLB) led to widescale usage with national program oversight and reimbursement. However, little is known about the success of these DPP-translation programs across subgroups of sociodemographic factors. This current effort investigated potential disparities in DPP-translation program primary goal achievement (physical activity and weight) by key sociodemographic factors.

**Methods:**

Data were combined from two 12-month community-based DPP-GLB trials among overweight/obese individuals with prediabetes and/or metabolic syndrome. We evaluated change in weight (kilograms and percent) and activity (MET-hrs/week) and goal achievement (yes/no; ≥5% weight loss and 150 min per week activity) after 6 and 12 months of intervention within and across subgroups of race/ethnicity (non-Hispanic white, non-Hispanic black), employment status, education, income, and gender.

**Results:**

Among 240 participants (85%) with complete data, most sociodemographic subgroups demonstrated significant weight loss. However, non-Hispanic white lost more weight at both 6 and 12 months compared to non-Hispanic black participants [median weight loss (IQR), 6 months: 5.7% (2.7–9.0) vs. 1.5% (1.2–7.5) *p* = .01 and 12 months: 4.8% (1.1–9.6) vs. 1.1% (− 2.0–3.7) *p* = .01, respectively]. In addition, a larger percentage of non-Hispanic white demonstrated a 5% weight loss at 6 and 12 months. Employment was significantly related to 12-month weight loss, with retired participants being the most successful. Men, participants with graduate degrees, and those with higher income were most likely to meet the activity goal at baseline and 12 months. Differences in physical activity goal achievement across gender, education, and income groups were significant at baseline, attenuated after 6 months, then re-emerged at 12 months.

**Conclusions:**

The DPP-GLB was effective in promoting weight loss and helped to alleviate disparities in physical activity levels after 6 months. Despite overall program success, differences in weight loss achievement by race/ethnicity were found and disparities in activity re-emerged after 12 months of intervention. These results support the need for intervention modification providing more tailored approaches to marginalized groups to maximize the achievement and maintenance of DPP-GLB behavioral goals.

**Trial registration:**

NCT01050205, NCT02467881.

## Background

An estimated 10.5% of the US adult population has diabetes, the majority of which is type 2 diabetes [1]. Various estimates demonstrate disproportionately higher diabetes prevalence in lower income and education groups [2, 3] and in non-Hispanic black individuals compared to non-Hispanic white (16.4% vs. 11.9% [1]). Diabetes is associated with complications such as cardiovascular disease, end-stage renal disease, and premature mortality, [4] which are also disproportionately prevalent among many of these same sociodemographic groups [5, 6] who are likely disadvantaged due to lower income and education, discrimination, and related social factors [3]. Thus, approaches to diabetes prevention that are effective across sociodemographic subgroups are a priority.

The landmark Diabetes Prevention Program (DPP) demonstrated that a behavioral lifestyle intervention was highly effective in decreasing or delaying the onset of type 2 diabetes among a diverse cohort of overweight/obese individuals with prediabetes [7]. Due to the success of translated versions of the DPP lifestyle intervention, the US Centers for Disease Control and Prevention (CDC) established a system for widescale dissemination of the intervention program [8]. Relatively recently, the Centers for Medicare and Medicaid Services (CMS) began offering reimbursement for CDC approved DPP-based lifestyle intervention [9]. With widespread implementation of DPP-based lifestyle intervention programs, it is important to ensure reimbursable programs are effective among sociodemographic groups who carry a disproportionate burden of type 2 diabetes.

The DPP-Group Lifestyle Balance (DPP-GLB), one of the CDC recognized/CMS reimbursed DPP-based lifestyle interventions, has shown to be effective in a variety of diverse community settings [10–15]. However, identifying potential disparities in uptake and maintenance of recommended behavioral changes during the course of the DPP-GLB would be beneficial in directing efforts to ensure equitable intervention effectiveness. It is also worth considering the potential for the DPP-GLB intervention to address disparities in health behaviors that may have existed at intervention baseline [3]. Identifying potential disparities in achieving the primary behavioral goals of the DPP-GLB could inform the need to modify the current intervention with possible solutions that include additional support and resources to promote success among disadvantaged sociodemographic groups. A better understanding of disparities in lifestyle intervention progress could also speak to the upstream influence of social determinants of health [16].

The primary goals of the DPP-GLB are the same as the DPP itself, to achieve a 7% weight loss (with a 5% weight loss accepted as the criteria for program success in these translation efforts; per CMS protocol [8, 9]), and to reach 150 min per week of moderate or greater intensity physical activity [7, 17]. We evaluated success in reaching these primary intervention goals across sociodemographic subgroups during a community-based DPP-GLB program.

## Methods

For this current effort, we combined participant data from two clinical trials offering a community-based DPP-GLB intervention, the Healthy Lifestyle Project (GLB-Healthy, 2010–2014) [12] and The Physical Activity and Sedentary Behavior Change study (GLB-Moves, 2014–2019). Both studies received University of Pittsburgh Institutional Review Board approval and all subjects provided written informed consent.

### Study population

Eligible participants were ≥ 18 years of age (GLB-Healthy) or ≥ 40 years of age (GLB-Moves), with a BMI > 24 kg/m^2^ (> 22 kg/m^2^ for Asian persons, consistent with the DPP BMI eligibility criteria [7]), and evidence of prediabetes defined as fasting glucose ≥ 100 to < 126 and/or hemoglobin A_1c_ 5.7–6.4%, and/or metabolic syndrome defined by National Cholesterol Education Program Adult Treatment Panel III criteria or hyperlipidemia and one component of metabolic syndrome [18]. Potential participants were ineligible if they had plans to move away in the 18 months following study enrollment, had diagnosed diabetes, were taking Metformin, had a recent (past 3 months) initiation or change in blood pressure or lipid medication, or were pregnant or breastfeeding.

All participants included in this effort, a total eligible sample of *n* = 282, received the intervention program in community sites with the primary intervention goals of a 7% weight loss and to reach or maintain 150 min or more of moderate or greater intensity physical activity each week. Participants in GLB-Healthy who received the intervention through a worksite, and GLB-Moves participants who were randomly assigned to receive an alternative physical activity goal were not included in the sample for the current analyses.

Study investigators partnered with community organizations in Allegheny County, PA (the greater Pittsburgh area) to offer the DPP-GLB program, including clinic assessment and the lifestyle intervention, in community sites. Recruitment efforts targeted community center members and individuals living in close proximity to the centers.

### Study design

The DPP-GLB is a yearlong behavioral lifestyle intervention program consisting of a core curriculum of 12 weekly sessions followed by four biweekly sessions during the first 6 months, and monthly “maintenance” sessions offered in the second 6 months. Study sessions consisted of small groups meeting in community centers for about an hour each session. The intervention design was identical across both studies. The GLB-Healthy and GLB-Moves were both randomized clinical trials with delayed intervention control groups, however all participants included in this effort eventually received the exact same yearlong DPP-GLB program. For the purpose of this analysis and in order to evaluate the DPP-GLB as it is offered and recognized by the CDC, measures from the clinic visits immediately preceding receiving the intervention curriculum and at 6 and 12 months of intervention were examined.

The DPP-GLB curriculum promotes balanced, calorie restricted eating to promote weight loss goals, strategies to increase and maintain moderate or greater intensity activity, and behavioral strategies to support these behavior modifications. All sessions were led by health coaches, who were health professionals such as nurses and dietitians trained to provide the GLB program. Health coaches were not involved in clinic assessments. Participants received additional support in the form of tracking tools, weigh-ins at in-person sessions, and group discussion.

Participants who were unable to attend any given in-person session were offered the opportunity to make up the sessions with the health coach, which included review and discussion of session materials.

### Measures

Sociodemographic measures including gender, race/ethnicity, education, and employment status were collected using demographic questionnaires completed during screening. A verbally administered form included the following prompts: “What is your gender?”, “Which of these racial categories best describe you?” (categories included: white, black/African American, American Indian or Alaskan Native, Asian, Pacific Islander, Other), and “Please tell me your ethnicity. Are you Hispanic, Latino or of Spanish origin?”. An additional form completed by the participants included the prompts: “Currently employed?” [possible responses: Working full-time (35 h or more/week), Working part-time (< 35 h/week), Unemployed or laid off and looking for work, Unemployed and not looking for work, Homemaker, Retired, Student, Disabled/unable to work, Other] and “Education” [8th grade or less, Some high school, High school graduate or GED, Some college or technical school, College graduate (bachelor’s degree), Graduate degree]. Income was estimated as the median income for the census tract of the participants home address using 2016 American Community Survey 5-year estimates [19].

Body weight was measured during clinic assessment visits at intervention baseline and at 6 and 12 months of intervention using the average of two measures taken with calibrated digital scales. We evaluated percentage weight loss as a continuous variable. Although the participant weight loss goal was 7%, the study goal itself was 5%, which is consistent with the threshold for success per CDC recognition of DPP-translation efforts and CMS reimbursement protocol [8, 9]. Thus we also examined a dichotomous “yes/no” success variable with the cut point of 5% indicating success.

Physical activity was measured using the Modifiable Activity Questionnaire, which has shown to be valid and reliable in capturing moderate or greater intensity leisure physical activity in adults [20, 21] and quantified in Metabolic Equivalent of Task (MET) hours per week. Total weekly leisure activity of ≥7.5 MET hours/week is considered roughly equivalent to the 150 min/week moderate or greater intensity activity goal. We evaluated physical activity level as a continuous variable and as a “yes/no” success variable with the cut point of ≥7.5 MET hours/week indicating success.

### Analysis

We excluded participants with missing weight, physical activity, or sociodemographic measures from the analysis (*n* = 43). We evaluated the distribution of participants within each sociodemographic category. Individuals (*n* = 10) reporting race/ethnicity other than non-Hispanic white (NHW) or non-Hispanic black (NHB) were not included in the primary analyses due to small subgroup sizes. Other categories of employment were combined (unemployed looking and not looking for work, homemaker, disabled/unable to work; no participants reported being a student). Income was categorized by distribution quartiles.

Significant differences across sociodemographic subgroups in the proportion of participants with weight loss success (5% loss, yes/no) at 6 and 12 months were evaluated using Fisher’s Exact or Chi-Square tests. Using these same tests, we evaluated differences across sociodemographic subgroups in the proportion of participants meeting the physical activity goal of ≥7.5 MET hours/week (yes/no) at baseline, 6 months and 12 months.

Absolute weight loss in kilograms at 6 and 12 months was calculated as the difference from baseline in measured weight at either 6 or 12 months, respectively. Percent weight loss was the weight loss at 6 or 12 months divided by the baseline weight. Change in physical activity was calculated as the difference in measured activity in MET hours/week from baseline to either 6 or 12 months. Due to the non-normal distribution of the change variables, we evaluated meaningful continuous change within each sociodemographic group using Wilcoxon Signed-Rank tests and between subgroups within any given sociodemographic factor using Wilcoxon Two-Sample or Kruskal-Wallis tests.

We also used Chi-square and Fishers Exact tests to evaluate independence between sociodemographic variables. Due to lack of independence between some sociodemographic variables and model fit restrictions related to the small number of participants within several sociodemographic subgroups, multiple regression analyses estimating the odds of weight loss success or physical activity were not performed. All analyses were conducted in SAS version 9.4 (SAS Inc. Cary, NC).

## Results

A total of 240 participants (85%) had complete data available at baseline and after 6 and 12 months of intervention. As shown in Table 1, 74.2% of participants were women and the mean age of participants was 62.5 years. The majority of participants (93% of those included in race/ethnicity analyses) identified as NHW. Most participants were retired (42.5%), and all but 12.5% had completed at least some college education. Baseline weight and activity across sociodemographic factors are also shown in Table 1.

**Table 1:** Baseline Demographic Data (*n* = 240), Weight and Activity

Characteristic	N (%) or mean (SD)	Baseline Weight (kg), Median (IQR)	Baseline Activity (MET hr/wk), Median (IQR)
***Age***	62.5 (10.2)	–	–
***Gender***			
Men	62 (25.8)	100.6 (92.2–118.9)	18.1 (6.0–29.4)
Women	178 (74.2)	88.3 (78.3–100.7)	9.1 (2.9–18.2)
***Race/ethnicity***^***a***^			
Non-Hispanic white	214 (93.0)	90.8 (80.2–105.8)	12.0 (3.8–21.3)
Non-Hispanic black	16 (7.0)	94.1 (87.5–113.5)	5.7 (0.0–17.3)
***Employment***			
Working full-time	91 (37.9)	100.6 (86.2–113.7)	14.0 (4.3–22.0)
Working part-time	30 (12.5)	87.0 (77.5–93.0)	7.5 (1.5–29.4)
Retired	102 (42.5)	88.4 (77.1–100.1)	12.2 (5.3–21.4)
Other employment status	17 (7.0)	89.8 (85.1–99.7)	7.9 (3.5–19.4)
***Education***			
High school graduate or less	30 (12.5)	85.5 (73.4–90.5)	9.8 (2.3–28.0)
Some college	78 (32.5)	91.0 (84.0–104.2)	8.0 (2.6–17.5)
College graduate	68 (28.3)	97.0 (84.4–111.2)	11.0 (3.3–18.4)
Graduate degree	64 (26.7)	91.8 (77.9–110.1)	17.0 (8.3–28.8)
***Annual Income (by home address census tract, in US dollars)***			
< 51,934	56 (23.3)	95.6 (80.0–110.2)	7.9 (3.3–17.2)
51,934 – < 65,105	58 (24.2)	90.5 (79.5–113.0)	7.0 (1.7–18.1)
65,105 – < 74,935.5	66 (27.5)	91.4 (82.8–99.7)	15.9 (8.5–28.8)
≥ 74,935.5	60 (25.0)	87.7 (78.0–110.2)	13.4 (3.5–21.3)

Allegheny County, PA. USA. Study date: 2010–2019. Eligible population: overweight with prediabetes and/or metabolic syndrome. a: Not included in analysis: Asian (*n* = 2), Hispanic white (*n* = 2), Hispanic black or African American (*n* = 1), Hispanic other (*n* = 1), non-Hispanic Multi/Other (*n* = 4). Percentages are of sample included (*n* = 230 for race/ethnicity)

Tests for independence of sociodemographic variables found that race/ethnicity and income (*p* < .001), employment and education (*p* < .001), and income and education (*p* = 0.03) were not independent. All other relationships between sociodemographic variables did not deviate from independence. Overall median attendance was 21 [interquartile range (IQR) 17–22] sessions, out of a possible 22, with a median of 20 or higher for each sociodemographic subgroup.

### Weight loss success

**Fig. 1:**
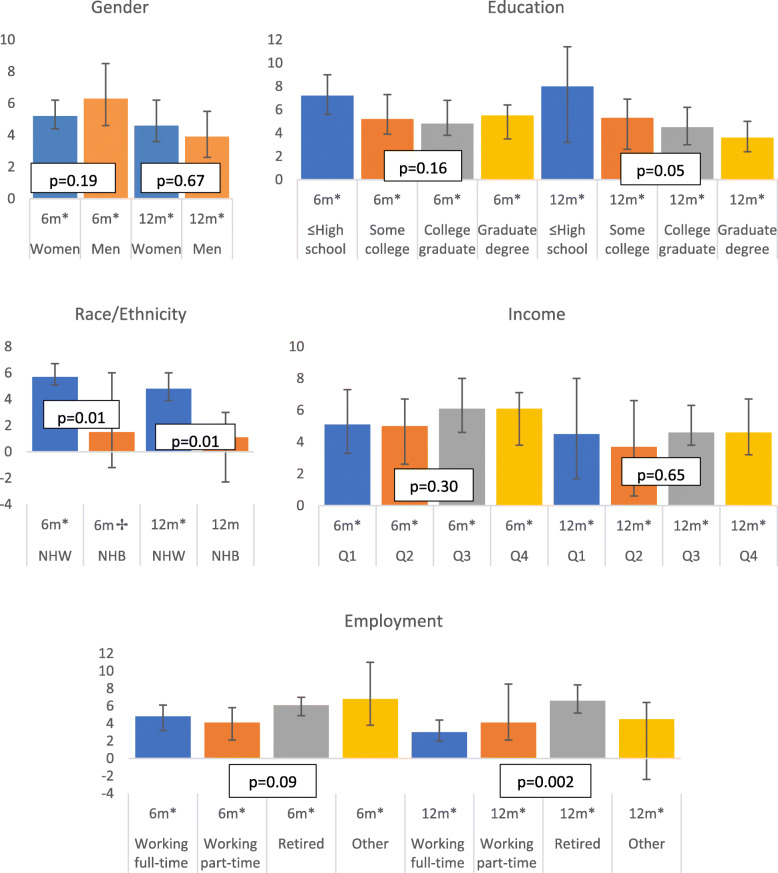
Weight Loss (%) as median with 95% confidence intervals at 6 and 12 months by Gender, Education, Race/Ethnicity, Income, and Employment. Legend: Allegheny County, PA. USA. Study date: 2010–2019. Eligible population: overweight with prediabetes and/or metabolic syndrome. *p < .05, ✢ *p* < .10 using Wilcoxon Signed-Rank Test of within-group change; Text box *p*-values determined using Wilcoxon Two Sample or Kruskal-Wallis Test of between-group change. *n* = 240; Race/Ethnicity *n* = 230. m: months; Q1: <$51,934, Q2: 1934 – < 65,105, Q3: 65,105 – < 74,935.5, Q4:≥ 74,935.5 median household income in US dollars

Figure 1 depicts percentage weight loss across sociodemographic subgroups. Within-group weight loss was significant, as both a percentage and in kilograms, for all categories of income, education, and gender. No between-group differences were noted by income. There was a borderline significant difference in percent weight loss across education subgroups at 12 months only, with the greatest percent weight loss among those with a high school education or less (*p* = 0.05) Notably, 60% in this group (high school education or less) reported they were retired. While no difference across gender subgroups was seen for percent weight loss, absolute weight loss in kilograms was significantly greater among men compared to women at 6 months (7.4 kg vs 4.7 kg weight loss respectively, *p* = 0.01), but not at 12 months (3.9 kg vs 4.2 kg, *p* = 0.67).

NHW demonstrated significant within-group change in percent and absolute weight loss at 6 months [median (IQR): 5.7% (2.7–9.0) *p* < .001; 5.1 kg (2.4–8.8) *p* < .001)]. NHB also had significant absolute weight loss [median (IQR): 1.8 kg (− 1.0–7.6), *p* = 0.04] at 6 months; percent weight loss was marginally significant [median (IQR): 1.5% (− 1.2–7.5) *p* = 0.07]. At 12 months, percent and absolute weight loss among NHW remained significant [median (IQR): 4.8% (1.1–9.6) *p* < .001; 4.3 kg (1.1–8.6) *p* < .001)] but was no longer significant among NHB. Comparing the two groups, NHW lost significantly more weight, more than double that of NHB at both 6 and 12 months (*p* = 0.01 at both time points).

All employment groups demonstrated significant within-group percent and absolute weight loss at both 6 and 12 months. Differences across employment groups in both percent and absolute weight loss were significant at 12 months. At 12 months, retired participants demonstrated the largest percent weight loss [Median (IQR): 6.6% (2.2–10.7); 5.7 kg (2.1–9.7)] and participants working full time demonstrated the smallest percentage weight loss [Median IQR: 3.0% (0.3–6.8), 3.1 kg (0.3–7.5)].

There were no significant differences in meeting the 5% weight loss goal (yes/no) by gender, education, or income, Table 2. There were borderline and statistically significant differences in reaching the weight loss goal (yes/no) among NHW versus NHB at 6 and 12 months [57.5% NHW vs 31.3% NHB (*p* = 0.07) and 49.1% NHW vs 18.8% NHB (*p* = 0.02) demonstrating success, respectively]. Meeting the weight loss goal also differed significantly by employment status at 12 months (with 59.8% of retired, 34.1% working full time, 40% working part time, and 47.1% other employment status demonstrating success, *p* = .004).

**Table 2:** Weight Loss Success (≥5%) Across Sociodemographic Subgroups at 6 and 12 months (*n* = 240)

	6 months			12 months		
	≥5% Weight Loss, n(%)		***p***-value	≥5% Weight Loss, n(%)		***p***-value
	No (***n*** = 109)	Yes (***n*** = 131)		No (***n*** = 128)	Yes (***n*** = 112)	
***Gender***						
Women	85 (47.8)	93 (52.3)	0.21	94 (52.8)	84 (47.2)	0.78
Men	24 (38.7)	38 (61.3)		34 (54.8)	28 (45.2)	
***Race/ethnicity***^***a***^						
Non-Hispanic white	91 (42.5)	123 (57.5)	0.07	109 (50.9)	105 (49.1)	0.03
Non-Hispanic black	11 (68.8)	5 (31.3)		13 (81.3)	3 (18.8)	
***Employment***						
Working full-time	47 (51.7)	44 (48.3)	0.12	60 (65.9)	31 (34.1)	.004
Working part-time	17 (56.7)	13 (43.3)		18 (60.0)	12 (40.0)	
Retired	38 (37.3)	64 (62.7)		41 (40.2)	61 (59.8)	
Other employment status	7 (41.2)	10 (58.8)		9 (52.9)	8 (47.1)	
***Education***						
High school graduate or less	9 (30.0)	21 (70.0)	0.25	12 (40.0)	18 (60.0)	0.19
Some college	37 (47.4)	41 (52.6)		38 (48.7)	40 (51.3)	
College graduate	35 (51.5)	33 (48.5)		39 (57.4)	29 (42.6)	
Graduate degree	28 (43.8)	36 (56.3)		39 (60.9)	25 (39.1)	
***Annual Income (by home address census tract, in US dollars)***						
< 51,934	27 (48.2)	29 (51.8)	0.73	30 (53.6)	26 (46.4)	0.99
51,934 – 65,105	29 (50.0)	29 (50.0)		32 (55.2)	26 (44.8)	
65,105 – 74,935.5	27 (40.9)	39 (59.1)		35 (53.0)	31 (47.0)	
> 74,935.5	26 (43.3)	34 (56.7)		31 (51.7)	29 (48.3)	

Allegheny County, PA. USA. Study date: 2010–2019. *P*-values determined using Chi Square or Fishers Exact Test for Differences of Proportions. Eligible population: overweight with prediabetes and/or metabolic syndrome. a: Race/ethnicity category *n* = 230, 6-month success no: *n* = 102, yes *n* = 128; 12-month success no: *n* = 122, yes: *n* = 108

### Physical activity success

Participants did not have to be inactive to participate in the DPP-GLB and may have met the physical activity goal at baseline. Therefore, we considered success in meeting the activity goal at baseline, 6 and 12 months. Given that, the percentage meeting the activity goal improved across all sociodemographic categories due to intervention at 6 and 12 months.

**Fig. 2:**
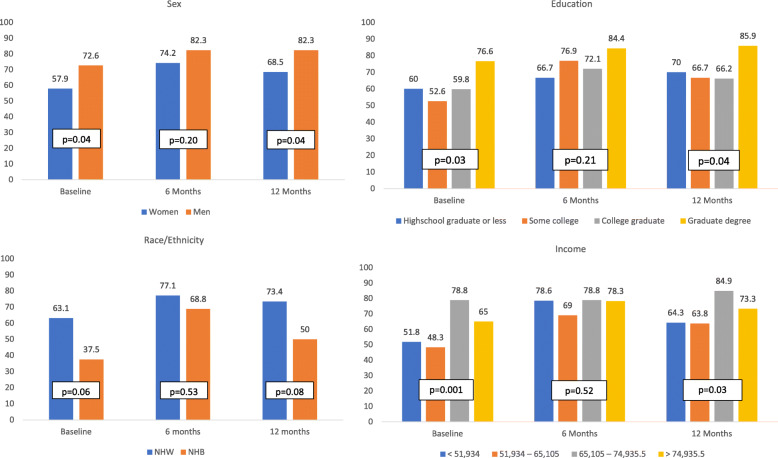
Physical Activity Success (% meeting ≥7.5 MET-hours/week goal) at Baseline, 6, and 12 months. Legend: Allegheny County, PA. USA. Study date: 2010–2019. Eligible population: overweight with prediabetes and/or metabolic syndrome. *P*-values determined using Chi Square or Fishers Exact Test for Differences of Proportions meeting goal vs not meeting goal across sociodemographic subgroups; Income measured in quartiles of median annual household income in US dollars. Employment data not shown, no significant differences noted. *n* = 240; Race/Ethnicity *n* = 230

In general, there were significant differences in meeting the physical activity goal (yes/no) across gender, education, and income subgroups at baseline that were no longer significant at 6 months but re-emerged at 12 months (Fig. 2). A significantly larger percentage of men relative to women met the physical activity goal at baseline (72.6% vs. 57.9%, *p* = 0.04) and at 12 months (82.3% vs. 68.5%, *p* = 0.04). Regarding education, the largest percentage of participants with a graduate degree or higher met the activity goal at each time point. Participants with a high school education or less showed continued improvement through the course of the intervention (60.0, 66.7, 70.0% at baseline, 6 and 12 months), a group that was more likely to not be working. Across categories of income, fewer participants in the lower income quartiles met the activity goal at baseline and at 12 months compared to higher income quartiles (baseline: 51.8, 48.3, 78.8, 65% *p* = .001 and 12 months: 64.3, 63.8, 84.9, 73.3% *p* = 0.03, lowest to highest quartiles).

Activity appeared significantly higher among NHW vs NHB at baseline (63.1% vs 37.5%, *p* = 0.06), however differences at 6 and 12 months were not significant. There were no significant differences at baseline, 6, or 12 months by employment (data not shown).

Change in physical activity from baseline to 6 months differed significantly by education and income subgroups, respectively (Table 3). Participants with some college education demonstrated the greatest median increase in physical activity of 9.6 (IQR: − 1.3–26.2) MET-hrs/wk., while those with a graduate degree or more demonstrated the smallest median increase of 1.2 (IQR: − 5.6–10.7) MET-hrs/wk. By income, median (IQR) MET-hours/week change from baseline to 6 months was largest in the lowest and highest quartiles [<$51,934: 7.4 (− 0.6–22.7), $51,934–65,105: 5.8 (0.0–22.0), $65,105–74,935.5: 1.2 (− 5.9–12.0), >$74,935.57.6 (− 6.2–19.4)]. At 12 months, change in physical activity from baseline was no longer significant between groups for education or income. Change was not significant at either time point between groups for gender, race/ethnicity, or employment.

**Table 3:** Continuous Physical Activity Change (in MET hours/week) as Median (IQR) at 6 and 12 months

	6 months			12 months		
	PA Change (MET hr/wk)	Within group ***p***-value^**b**^	Between Group ***p***-value^**c**^	PA Change (MET hr/wk)	Within group ***p***-value^**b**^	Between Group ***p***-value^**c**^
***Gender***						
Women	5.2 (−2.3, 17.4)	< 0.01	0.92	3.0 (−3.0, 10.6)	< 0.01	0.61
Men	5.5 (−5.4, 22.9)	< 0.01		1.3 (−7.0, 14.3)	0.18	
***Race/ethnicity***						
Non-Hispanic white	5.3 (− 3.7, 18.3)	< 0.01	0.76	3.0 (− 3.9, 12.3)	< 0.01	0.76
Non-Hispanic black	5.9 (0.1, 26.2)	0.01		1.53 (−3.9, 11.4)	0.42	
***Employment***						
Working full-time	4.1 (−3.7, 14.6)	< 0.01	0.63	2.9 (−2.8, 12.4)	< 0.01	0.36
Working part-time	4.5 (−1.3, 14.4)	0.11		0.2 (−7.4, 7.2)	0.71	
Retired	7.4 (−2.9, 22.1)	< 0.01		2.5 (−2.7, 12.4)	< 0.01	
Other employment status	5.3 (−1.7, 13.2)	0.17		7.7 (−7.6, 15.8)	0.28	
***Education***						
High school graduate or less	4.8 (−6.3, 20.8)	0.14	0.02	0.6 (−7.2, 9.9)	0.34	0.77
Some college	9.6 (−1.3, 26.2)	< 0.01		2.8 (−1.5, 14.2)	< 0.01	
College graduate	6.4 (−2.0, 14.3)	< 0.01		1.1 (−4.5, 10.4)	0.09	
Graduate degree	1.2 (−5.6, 10.7)	0.29		3.8 (−6.5, 11.7)	0.07	
***Annual Income (by home address census tract, in US dollars)***						
< 51,934 (*n* = 56)	7.4 (−0.6, 22.7)	< 0.01	0.04	5.1 (−3.9, 14.3)	0.01	0.83
51,934 – < 65,105 (*n* = 58)	5.8 (0.0, 22.0)	< 0.01		2.0 (− 0.6, 7.4)	0.01	
65,105 – < 74,935.5 (*n* = 66)	1.2 (−5.9, 12.0)	0.31		3.4 (−7.3, 12.6)	0.16	
≥ 74,935.5 (*n* = 60)	7.6 (−6.2, 19.4)	0.01		1.3 (−4.3, 14.2)	0.06	

Allegheny County, PA. USA. Study date: 2010–2019. a: Race/ethnicity category *n* = 230; b: Wilcoxon Signed-Rank Test; c: Wilcoxon Two-Sample Test or Kruskal-Wallis Test

## Discussion

As a whole, the DPP-GLB lifestyle intervention was effective in helping participants across most sociodemographic groups lose weight and meet their physical activity goals. However, despite widespread success due to intervention participation, disparities existed in the amount of weight lost between groups with, for example, NHW showing greater weight loss than NHB participants. We also found that retired participants had relatively greater weight loss than other employment groups at the end of the maintenance phase of the intervention. In addition, it appeared that the intervention may have begun to help equalize meeting the physical activity goal by 6 months among women, NHB, and those with lower income/education, all who had lower activity levels at baseline. Unfortunately, the disparities in activity levels found at baseline and attenuated at 6 months began to emerge again by 12 months. These findings identify the need for more effective, individualized approaches during the intervention maintenance phase such as offering additional support in order to address the complex influence of existing sociodemographic factors that impact the maintenance of weight loss and increased physical activity.

Our race/ethnicity weight loss results are in line with findings from the DPP efficacy trial and in DPP-based lifestyle intervention community translation efforts. In the DPP multicenter clinical trial, white relative to black participants had more success with weight loss after the 6 month core and at the end of the formal trial, after around 3 years [22]. In CDC recognized DPP-translation programs, NHB individuals were less likely to achieve a 5% weight loss compared to NHW [23]. Studies exploring weight loss success in DPP-translation efforts in black populations have also found suboptimal weight loss [14, 24, 25]. This consistent finding of less weight loss among NHB participants compared to NHW emphasizes the need to modify existing DPP-based lifestyle interventions to enhance effectiveness among NHB individuals.

In the DPP clinical trial, there were no overall gender differences in the odds of meeting the weight loss goal at either the end of the core intervention or at study end [22] although black women had significantly smaller weight loss compared to other racial/ethnic groups [26]. Unlike the DPP clinical trial, DPP-translation findings have found a higher odds of meeting a 5% weight loss goal among men compared to women [8]. However, similar to the DPP clinical trial, translation efforts have echoed smaller weight loss among black women [24]. In our current effort, men lost more weight, in kilograms, than women at 6 months although we had too small a sample size to look at goal success by race/ethnicity*gender.

Significant weight loss differences were found by employment and education in this effort. In the DPP multicenter clinical trial, weight loss success differed significantly by employment, with retired participants demonstrating the highest percentage who were successful after the full intervention [22]. Our findings also demonstrated a relatively high weight loss success rate among retired participants. In regard to education, which was significantly correlated with employment in this current effort, our study suggested differences by education at 12 months, with the most success being among the “high school or less” group. Notably, the majority (70%) of the high school education group also had a non-working employment status, with most retired, which may have contributed to their weight loss success. These findings are in contrast to those in a combined sample of CDC recognized DPP-translation efforts in which there were not significant differences in weight loss success by education [8].

Weight loss success by income in the DPP clinical trial and DPP translation literature is inconsistent. Participants from lower income groups showed the highest weight loss success in the DPP trial, [22] although translation efforts have indicated less weight loss among some low income groups [27]. In contrast to employment and education, our findings showed no weight loss differences by income.

While weight loss success has been evaluated in a number of DPP-translation efforts, success in physical activity is less frequently documented [28]. In the DPP itself, Hispanic American, Native American and Asian American individuals, retired participants, low-income participants, and men were most likely to meet the physical activity goal, respectively, after the full intervention [22]. Our findings demonstrated that fewer NHB participants appeared to have met the activity goal compared to NHW at 12 months. Unlike the DPP clinical trial, participants with higher incomes were most likely to meet activity goals in our translation findings. Similar to the DPP clinical trial, we also saw a higher percentage of men meeting the activity goal at baseline and at 12 months.

To our knowledge, we are the first study to evaluate sociodemographic disparities in physical activity in a DPP-translation effort. The lack of significant differences in physical activity by any sociodemographic category at 6 months and general improvement in the percentage meeting the activity goal during the course of the intervention indicate that the intervention was successful early on in promoting increased activity among those most in need of improvement. More research is warranted regarding how to sustain the improvements found at 6 months into the maintenance phase of the intervention and beyond.

The driving factors for differences in weight loss and physical activity across sociodemographic subgroups are likely multifaceted and complex. Social determinants of health that lead to disadvantage among certain sociodemographic groups have been indicated to increase risk for diabetes and related cardiometabolic conditions [29–31]. These social determinants likely contributed to differences observed in physical activity at baseline in this effort, and may have contributed to differences in behavior change and change maintenance during the course of the DPP-GLB intervention. Ongoing research into the upstream factors contributing to disadvantage (e.g. exposure to adversity, food security, built environment, etc.) and additional efforts to address these factors are essential pieces of the puzzle in promoting equitable approaches to disease prevention [29, 31].

This effort has several strengths. We were able to evaluate success in achieving physical activity and weight loss goals in a community DPP-based lifestyle intervention program offered over a span of almost a decade. Participants provided overwhelmingly positive feedback of the intervention, as has been previously reported [12], and demonstrated excellent attendance, with no meaningful differences in attendance across sociodemographic groups. Engaging intervention participants in their local communities likely contributed to excellent program attendance. Close collaboration with community partners also likely contributed in part to the successful implementation of this community-based DPP-GLB program. Finally, the DPP-GLB also offers a CDC recognized curriculum that is intentionally adaptable to individual needs and preferences, making it an ideal program for use across a variety of diverse settings.

While every effort was made to include participants across the socioeconomic spectrum and among a variety of communities, this effort is limited by the amount of racial/ethnic diversity in the study sample, likely due to the lack of diversity in the greater Pittsburgh area. Also, the fact that physical activity level was not part of the eligibility criteria allowed for some participants to be relatively active at baseline. Finally, income was defined using neighborhood level estimates, which may not directly reflect individual level income. It is possible that additional neighborhood level factors may also influence success in lifestyle intervention programs.

## Conclusion

The DPP-GLB is an appealing intervention option with the potential to improve risk factors for developing type 2 diabetes and related metabolic conditions. In fact, there have already been a variety of cultural adaptations of the DPP lifestyle intervention to suit a diverse array of populations and settings [32–37]. In agreement with such efforts, our current findings suggest that NHB and non-retired participants would benefit from additional support in achieving and maintaining weight loss, while women, NHB, lower education and lower income participants would benefit from additional support in maintaining adequate activity levels. Alongside population level approaches to promote health equity, additional adaptations of DPP-based lifestyle intervention programs, particularly during the maintenance phase when intervention contact is less frequent, should be considered to minimize these disparities in weight loss and physical activity found at program end.

## Data Availability

The datasets used and/or analyzed during the current study are available from the corresponding author on reasonable request.

## Abbreviations

DPP: Diabetes prevention program
GLB: Group lifestyle balance
CDC: US Centers for disease control and prevention
CMS: Centers for medicare and medicaid services
MET: Metabolic EQUIVALENT OF TASK
NHW: Non-hispanic white
NHB: Non-hispanic black
